# HKT sodium and potassium transporters in *Arabidopsis thaliana* and related halophyte species

**DOI:** 10.1111/ppl.13166

**Published:** 2020-07-23

**Authors:** Akhtar Ali, Natalia Raddatz, Jose M. Pardo, Dae‐Jin Yun

**Affiliations:** ^1^ Institute of Glocal Disease Control Konkuk University Seoul 05029 South Korea; ^2^ Department of Biomedical Science & Engineering Konkuk University Seoul 05029 South Korea; ^3^ Instituto de Bioquímica Vegetal y Fotosíntesis, cicCartuja, CSIC‐Universidad de Sevilla Americo Vespucio 49, Sevilla 41092 Spain

## Abstract

High salinity induces osmotic stress and often leads to sodium ion‐specific toxicity, with inhibitory effects on physiological, biochemical and developmental pathways. To cope with increased Na^+^ in soil water, plants restrict influx, compartmentalize ions into vacuoles, export excess Na^+^ from the cell, and distribute ions between the aerial and root organs. In this review, we discuss our current understanding of how high‐affinity K^+^ transporters (HKT) contribute to salinity tolerance, focusing on HKT1‐like family members primarily involved in long‐distance transport, and in the recent research in the model plant Arabidopsis and its halophytic counterparts of the *Eutrema* genus. Functional characterization of the salt overly sensitive (SOS) pathway and HKT1‐type transporters in these species indicate that they utilize similar approaches to deal with salinity, regardless of their tolerance.

**Abbreviations**
Asn: asparagine
Asp: aspartic acid
CBL: calcineurin B‐like
CIPK: CBL‐interacting protein kinase
HKT: high‐affinity K^+^ transporter
Ser: serine
SOS: salt overly sensitive

## Introduction

Among abiotic stresses, soil salinity constitutes a major factor in reducing crop productivity and yield (Munns et al. 2012, Zorb et al. 2019). The accumulation of salt in the soil of areas used for crop production, often irrigated with underground water, has developed into a major problem, limiting the growth and productivity of many important crops. The majority of crop plants are salt sensitive and unable to adapt to the stress factors associated with the increasingly elevated levels of salts in the soil, including ionic, osmotic and oxidative stresses, resulting in economic losses and societal disruptions. In saline soils, the ability of plants to grow, flower and develop seeds or fruits is severely compromised in a concentration‐dependent manner. As a consequence, there is a strong interest in studying mechanisms of salinity tolerance in plants.

High salinity leads to osmotic stress, sodium ion‐specific toxicity, nutritional deficiencies and oxidative stress (Shabala 2013, Zhu 2016, Isayenkov and Maathuis 2019) with inhibitory effects on physiological (e.g. photosynthesis inhibition), biochemical (e.g. protein stability and quantity) or developmental (e.g. retarded flowering) pathways (Li et al. 2007, Chaves et al. 2009, Kim et al. 2013, Hanin et al. 2016, Silveira and Carvalho 2016). For most plant species, Na^+^ toxicity is the main growth inhibitory factor (Munns 2002) at least in part because of the physicochemical similarities of Na^+^ with the macronutrient potassium (K^+^), which results in the displacement of K^+^ from the cellular milieu. However, Na^+^ ions, due to their rigid hydration shells compared to K^+^, has limited ability to replace K^+^ as the coordinating ion of proteins (Benito et al. 2014). Consequently, plants try to maintain a low Na^+^/K^+^ ratio in their cytosol, and the magnitude of this ratio is often used as a proxy to estimate the intensity of the salinity stress and the salt tolerance of plants (Shabala and Pottosin 2014). To cope with increased Na^+^ in soil water, plants have evolved biochemical resources and signaling pathways to restrict Na^+^ influx, re‐export to counteract the un‐avoidable influx of Na^+^, redistribute the ion through the plant organs via the xylem and to compartmentalize Na^+^ into vacuoles in various tissues (Qiu et al. 2002, Kim et al. 2007, Møller et al. 2009, Oh et al. 2009, Shabala 2013, Maathuis 2014). Moreover, K^+^ uptake in the presence of high external Na^+^ concentrations is equally crucial to preserve the supply of this essential nutrient (Assaha et al. 2017, Raddatz et al. 2020, Rubio et al. 2020). Na^+^ influx depolarizes the plasma membrane, leading to K^+^ efflux via depolarization‐activated outward rectifier K^+^ channels (Demidchik 2014). In addition, high cytoplasmic Na^+^ inhibited K^+^ uptake by the root AKT1 channel of Arabidopsis (Qi and Spalding 2004).

The main means by which plants can cope with excess Na^+^ are the salt overly sensitive (SOS) pathway, comprising an array of diverse signaling intermediaries and regulatory proteins that ultimately control the activity of SOS1, the leading plasma membrane Na^+^/H^+^ exchanger governing the efflux of Na^+^ in roots and loading into the xylem vessels for the long‐distance transport out of the roots. Counteracting the activity of SOS1 is the family of high‐affinity K^+^ transport (HKT) proteins, which despite their name are Na^+^ transporters that operate in the retrieval of Na^+^ from the xylem sap in both monocots and dicots (Class I HKTs), and that constitute a high‐affinity Na^+^ uptake pathway in the roots of monocots under K^+^ starvation (Class II; Horie et al. 2001, Sunarpi et al. 2005). There are excellent reviews dealing with these Na^+^ transport systems in glycophytes (Assaha et al. 2017), but much less is known in halophytes, a diverse group of plants highly tolerant to salinity (Flowers et al. 2010). In this review, we will summarize what is known about the SOS and HKT systems in halophytes and how the features of individual transporters may contribute to their salt tolerance. Emphasis will be given to comparative analyses between HKT1 orthologs from Arabidopsis and related halophytic species because structural and kinetic features of these proteins have been functionally related to salt tolerance.

## The SOS pathway

In genetic terms, the most significant mechanism that plants use to overcome sodicity is the SOS pathway (Ji et al. 2013, Assaha et al. 2017). The molecular and physiological analyses of the SOS pathway have been conducted on glycophytic species in which forward and reverse genetic resources are available, whereas the knowledge gained from halophytes is still scant (Oh et al. 2010, Shabala 2013). The core SOS pathway was initially described as the combined roles of three proteins, SOS3, a calcium‐binding protein, SOS2, a serine/threonine‐protein kinase and SOS1, a Na^+^/H^+^ antiporter (Qiu et al. 2002, Quintero et al. 2002). SOS1 is activated by the combined functions of SOS2 and SOS3, which form a complex that directly phosphorylates and activates the Na^+^/H^+^ exchange activity of SOS1 (Quintero et al. 2011). Further research has incorporated additional elements and functions to the SOS pathway (Ji et al. 2013). CBL10/SCaBP8 is an alternative CBL subunit of SOS2/CIPK24 that regulates SOS1 activity mainly in shoots, whereas SOS3/CBL4 seems to be more important in the roots of Arabidopsis (Quan et al. 2007). CBL10 also appears to regulate SOS1 activity in association with CIPK8 (Yin et al. 2020). CBL10 and SOS2 also interact at the tonoplast, suggesting that the complex may alleviate Na^+^ toxicity by modulating the sequestration of Na^+^ in vacuoles through unknown transporters (Kim et al. 2007, Yang et al. 2019). Two 14‐3‐3 proteins bind to and inhibit SOS2 upon phosphorylation of Ser‐294 by PKS5/CIPK11 (Zhou et al. 2014, Yang et al. 2019b). However, under salt stress, Ca^2+^‐activated 14‐3‐3 proteins repress PKS5, thereby releasing the inhibition of SOS2 (Yang et al. 2019b). Moreover, when active, PKS5 down‐regulates H^+^‐ATPase activity at the plasma membrane (Fuglsang et al. 2007) and salt‐dependent inactivation of PKS5 enhances the activity of H^+^‐ATPase and Na^+^/H^+^ exchange (Yang et al. 2019b). Activation of MPK6, upon the salinity‐induced synthesis of phosphatidic acid by phospholipase D (PLD), is also required for the full activity of SOS1 (Yu et al. 2010). The SOS pathway interacts with the regulation of carbon metabolism through the kinases GRIK1 and GRIK2 (a.k.a. SnRK1 activating kinases). GRIK kinases activate kinases SnRK1.1 and SnRK1.2 related to sugar metabolism, and also SOS2 under salt stress, thus, linking the regulation of cellular energy to salt tolerance (Barajas‐Lopez et al. 2018). Last, salinity alters the time of flowering and, surprisingly, regulators of flowering have an impact on salt tolerance. For instance, GIGANTIA (GI), a positive regulator in the photoperiodic branch controlling flowering time, sequesters SOS2 under non‐saline conditions. Upon salt stress and in a Ca^2+^‐dependent manner, GI is degraded, flowering is delayed, and SOS2 released from inhibition, which then supports salt tolerance through SOS1 (Kim et al. 2013). Deletion of *GI* promotes superior salt tolerance by derepressing the SOS pathway. By contrast, SOS2 has no function in setting flowering time, but SOS3 plays a positive role in flowering specifically under salt stress by an as yet unknown mechanism (Li et al. 2007, Kim et al. 2013).

The SOS pathway serves two important processes leading to salt tolerance. One is a cellular‐based mechanism that relies on the efflux of Na^+^ back to the apoplast or to the soil solution. The second but not least important function is the control of Na^+^ loading into the xylem, which has been confirmed in Arabidopsis, tomato and rice (Shi et al. 2002, Olias et al. 2009, El Mahi et al. 2019). The relative contribution of each of these two processes governed by the SOS proteins to the overall tolerance of the plant remains to be determined. It could be argued that the much dramatic salt‐sensitive phenotype of the Arabidopsis mutants lacking the single‐copy gene *SOS1* compared to *hkt1* mutants imply that Na^+^ efflux dominates over interorgan distribution. However, the function of SOS1 impinges in both processes, being the two of them defective in *sos1* mutants.

In line with the fundamental roles of the SOS pathway in salt tolerance, RNAi‐mediated knockdown lines of *SOS1* in *Eutrema salsuginea* (previously known as *Thellungiella salsuginea*), an Arabidopsis related halophyte, lead to loss of halophytism, which further strengthens the importance of SOS1 as an ubiquitous halotolerance determinant (Oh et al. 2009). The superior salt tolerance of *E*. *salsuginea* could not be linked to a more efficacious SOS1 protein, but to the enhanced expression of *SOS1* in both leaves and roots compared to Arabidopsis (Oh et al. 2009). Suppression of *SOS1* rendered *E*. *salsuginea* with a high shoot Na^+^‐accumulating phenotype, resulting from a lack of control over Na^+^ uptake and distribution via xylem. Na^+^ accumulated preferentially in the root xylem parenchyma of *SOS1*‐suppressed plants, displacing K^+^ from these cells (Oh et al. 2009). The function of SOS1 in the regulation of xylem loading also appears to be crucial for the salt‐accumulating halophyte *Salicornia* spp., in which the constitutively enhanced expression of *SOS1* in the root could be essential to maintain a constant flow of Na^+^ via the xylem to the shoot (Yadav et al. 2012, Katschnig et al. 2015).

Despite the essentiality of the SOS pathway in every species in which its contribution to salt tolerance has been analyzed, SOS components have not been identified in quantitative genetics and genomic analyses of salt tolerance, with the only exceptions of *SOS3/CBL4* in barley and wheat, and *SOS1* of wheat (Rivandi et al. 2011, Luo et al. 2017). This implies that there is little natural variability in the SOS system between related species and cultivars with contrasting salt tolerance. This is further supported by the evolutionary constraints observed in SOS1/NHX7 compared to other members of the NHX family from 32 plant species (Pires et al. 2013). By contrast, HKT, which also play crucial roles in the Na^+^ and K^+^ homeostasis under salt stress, have been identified numerous times as QTLs and GWAS being significantly associated with either high K^+^ and/or low Na^+^ content in shoots, and with salt tolerance (Ren et al. 2005, Hamamoto et al. 2015, Nieves‐Cordones et al. 2016, Henderson et al. 2018, Zhang et al. 2018, Cao et al. 2019). These findings, together with the salt‐sensitive phenotype often associated to loss‐of‐function mutants, show that HKT proteins represent a paramount line of defense that plants use against high salinity, and that a significant natural variability exists that can be utilized in breeding programs (Munns et al. 2012, Hamamoto et al. 2015). Therefore, we will devote the rest of this review to update our current understanding of HKT protein structure and function.

## Role of HKT transporters in salt tolerance

**Fig. 1:**
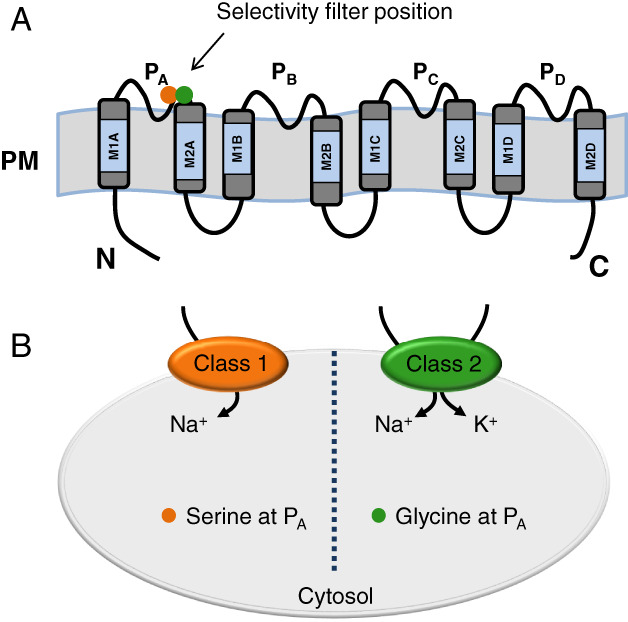
Structure and classification of HKT proteins. (A) Basic structure of the HKT proteins. HKTs have eight transmembrane domains (M1_A_–M2_D_) and four pore‐loop domains (P_A_–P_D_). Serine (blue) and glycine (green) residues responsible for Na^+^/K^+^ selection are indicated by arrows in the first pore‐loop region, which is known as the selectivity filter position. N and C indicate N‐ and C‐terminus of HKT proteins. (B) HKT1 proteins are divided into two classes, classes I and II. Most members of class I possess a serine residue in the selectivity filter position, whereas most class II transporters contain a glycine residue at the same position.

*HKT transporters* have been identified and widely characterized in model plants such as Arabidopsis and rice (Nieves‐Cordones et al. 2016). HKT proteins are formed by four repetitions, MPM, M1_A_‐P_A_‐M2_A_–M1_D_‐P_D_‐M2_D_, where “M” corresponds to the transmembrane segment and “P” to pore‐loop domain. The assembly of these repetitions forms a permeation pathway comparable to that of K^+^ channels. This structure was confirmed by the crystal structure of the K^+^ transporter TrkH from *Vibrio parahaemolyticus* (Cao et al. 2011). In three of the four pore‐loop domains, P_B_ to P_D_, all HKT proteins contain the GYG motif that is highly conserved in the selectivity filter of K^+^ transporting channels, but variability exists in the first position of this conserved motif in P_A_ (Fig. 1). The HKTs family has been divided into two classes based on a structural determinant located in the first P domain, P_A_. Class I members, found both in monocots and dicots, have a highly conserved serine in P_A_ and hence are also called the SGGG‐type (Maser et al. 2002b, Hauser and Horie 2010). These are ubiquitous in plants, Na^+^‐selective, and mostly promote the removal of Na^+^ from the xylem sap, which results in Na^+^ sequestration into xylem parenchyma cells (Maser et al. 2002a, Sunarpi et al. 2005). This mechanism prevents transport to, and accumulation of Na^+^ in the shoot over time and reduces Na^+^ toxicity by confining it to the roots, thus, protecting aboveground tissues from damage (Davenport et al. 2007). Numerous reports have shown that HKT1‐type transporters determine the balance between Na^+^ and K^+^ under salt stress (Ren et al. 2005, Hauser and Horie 2010, James et al. 2011, Ali et al. 2012, Oomen et al. 2012, Maathuis 2014, Véry et al. 2014, Wang et al. 2014, Jaime‐Pérez et al. 2017). In Class II members, found exclusively in monocots, the structural determinant of ion selectivity is composed of four glycine in the GYG motif of P_A_ to P_D_ (GGGG‐type; Maser et al. 2002b, Hauser and Horie 2010). Although they are permeable to K^+^, HKT2s can operate as Na^+^/K^+^ symporters (Fig. 1; Horie et al. 2001, Platten et al. 2006). Through 3D comparative modeling, Cotsaftis et al. (2012) proposed that the replacement of G–S in Class I members imposed a steric hindrance causing K^+^ to be transported unfavorably. Likewise, in Class II HKTs, the G would facilitate the transport of K^+^, without ruling out that under certain conditions, the transport of Na^+^ was also possible (Maser et al. 2002b). Some exceptions to this rule are EcHKT1;2 from *Eucalyptus camaldulensis*, McHKT1;1 from *Mesembryantemum crystallinum*, EsHKT1;2 from *Eutrema salsuginea* (previously *Thellungiella salsuginea* or *T*. *halophila*) or EpHKT1;2 from *Eutrema parvula* (also known as *Schrenkiella parvula*; previously *Thellungiella parvula*). These proteins transport K^+^, but they contain a Ser in the P_A_ domain (Fairbairn et al. 2000, Su et al. 2003, Jabnoune et al. 2009, Ali et al. 2012, Ali et al. 2018), which indicates that K^+^ permeability in HKTs depends on additional amino acids besides the Ser/Gly dichotomy in the pore. It is noteworthy that the HKT1 homologs departing from the S/G rule of ion selectivity are found mostly in either halophytes (e.g. EsHKT1;2 from *E*. *salsuginea* and McHKT1 from *Mesenbryanthemum crystallinum*) or salt‐tolerant glycophytes (EcHKT1;2 from *Eucalyptus camaldulensis*; Assaha et al. 2017), suggesting an evolutionary advantage of this polymorphism in providing stress tolerance.

In the case of Class II members, two rice (*Oryza sativa*) HKT transporters, OsHKT2;1, isolated from Nipponbare and OsHKT2;2 present in the salt‐tolerant Pokkali cultivar, have been amply characterized. These share high homology with 91% amino acid, and 93% cDNA sequence identity (Horie et al. 2001). However, in heterologous expression systems, they show differential Na^+^/K^+^ selectivity. OsHKT2;1 mainly transports Na^+^, whereas OsHKT2;2 mediates both K^+^ and Na^+^ uptake (Horie et al. 2001, Garciadeblas et al. 2003, Kader et al. 2006, Horie et al. 2007, Oomen et al. 2012). Unlike most members of Class II, OsHKT2;1 has a Ser residue instead of Gly in the P_A_ domain. The Na^+^ uptake of OsHKT2;1 highly correlated with the presence of this residue (Horie et al. 2001, Maser et al. 2002a, Garciadeblas et al. 2003). Data obtained with the loss‐of‐function mutant *oshkt2;1* suggest that OsHKT2;1 mediates a large Na^+^ influx into K^+^‐starved roots, compensating the lack of K^+^ (Horie et al. 2007). OsHKT2;1 can also mediate K^+^ transport, depending on the external concentration of both K^+^ and Na^+^ (Jabnoune et al. 2009, Yao et al. 2010). On the other hand, OsHKT2;2 has the typical GGGG domain like the member of the Class II HKT transporters. It is highly permeable to both K^+^ and Na^+^ in a wide range of concentrations, and functions preferentially as a symporter (Horie et al. 2001, Yao et al. 2010). Studies have reported the impact that K^+^ ions exert on Na^+^ transport in OsHKT2;2. However, this effect has only been seen at low K^+^ concentrations, diminishing with higher concentrations of extracellular K^+^ (Oomen et al. 2012, Riedelsberger et al. 2018). Thus, at millimolar Na^+^ concentrations and in the absence of K^+^, OsHKT2;2 facilitates the entry of Na^+^ (Horie et al. 2001, Kader et al. 2006, Oomen et al. 2012), but with an increase in the concentration of K^+^, the uptake of Na^+^ ions is reduced (Riedelsberger et al. 2018). Another interesting case is the natural variant, NoOsHKT2;2/1, identified in the highly salt‐tolerant rice cultivar NonaBokra (Oomen et al. 2012). This variant probably originated from a deletion in chromosome 6 producing a chimeric gene, where its 5′ region corresponding to the first three MPM domains, is homologous to OsHKT2;2, and more permeable to K^+^ (Horie et al. 2001). Its 3′ region corresponding to the last MPM domain is homologous with OsHKT2;1, weakly permeable to K^+^ and involved in root Na^+^ uptake (Horie et al. 2001, Golldack et al. 2002, Garciadeblas et al. 2003, Horie et al. 2007). NoOsHKT2;2/1 is essentially expressed in roots and shows a strong permeability for Na^+^ and K^+^, even at high external Na^+^ concentrations. Slopes of variation of NoOsHKT2;2/1 transport close to 20 mV per activity decade for both ions indicated that Na^+^ and K^+^ are the main ions transported, with a stoichiometry close to 1:1 (Jabnoune et al. 2009), contributing to salt tolerance in NonaBokra by enabling root K^+^ uptake under saline conditions (Oomen et al. 2012).

In wheat (*Triticum aestivum*), TaHKT2;1 seems to have a function similar to that of OsHKT2;1 (Horie et al. 2009). Preferentially expressed in the root cortex, this transporter has a role in root Na^+^ uptake induced by K^+^ deficiency, although it has also been reported to transport K^+^ (Schachtman and Schroeder 1994). In the same way, expression of *HvHKT2;1* of barley (*Hordeum vulgare)* is induced by K^+^ deficiency in roots and shoots and by high Na^+^ concentration in shoots. In *Xenopus* oocytes, HvHKT2;1 has a low affinity for Na^+^, a variable affinity for K^+^ that depends on the external Na^+^ concentration, and inhibition by K^+^ at about 5 mM (Mian et al. 2011, Hmidi et al. 2019). Transgenic barley lines over‐expressing HvHKT2;1 showed higher Na^+^ concentration in xylem, enhanced translocation of Na^+^ to shoots and higher Na^+^ accumulation in the leaves in comparison to the non‐transformed plants. Moreover, transgenic plants that grew in limiting K^+^ conditions, showed a significant increase in shoot K^+^ content. This indicates that HvHKT2;1 could be involved in the absorption or re‐absorption of K^+^ in the roots at very low concentrations of this cation (Mian et al. 2011). Recently, the transporter named HmHKT2;1 (*Hordeum maritimum*), homolog to HvHKT2;1, has been characterized (Hmidi et al. 2019). Electrophysiological analysis in *Xenopus* oocytes shows that HmHKT2;1 has a much higher affinity for both Na^+^ and K^+^ than HvHKT2;1 and a Na^+^/K^+^ symporter behavior in a very broad range of Na^+^ and K^+^ concentrations, due to reduced K^+^ blockage of the transport pathway. The analysis of chimeras between HvHKT2;1 and HmHKT2;1 allowed the identification of a region, composed of the fifth transmembrane segment, M1_C_ and the adjacent extracellular loop, P_C_, as a key domain in the determination of the affinity for Na^+^ and the level of K^+^ in HKT2;1 (Hmidi et al. 2019).

Oddly, HvHKT1;5 from barley may negatively affect the plant performance in a saline environment because RNAi‐mediated down‐regulation of *HvHKT1;5* resulted in salt tolerance instead of the anticipated sensitivity (Huang et al. 2020). HvHKT1;5 is a Na^+^‐specific transporter inhibited by external K^+^ and localized to the plasma membrane of root stele cells. Contrary to common findings, knocking down *HvHKT1*;*5* resulted in a decrease in the Na^+^ concentration in xylem sap and reduced Na^+^ translocation from roots to shoots, which would explain the increased salt tolerance of the transgenics compared with the wild‐type plants. These findings suggest that HvHKT1;5 is involved in Na^+^ loading to the xylem, which is opposite to the function of the HKT1;5 homologs expressed in the root stele of rice and wheat (Ren et al. 2005, Munns et al. 2012). An unexplained observation is that expression of *HvHKT1*;*5* increased under saline conditions (Huang et al. 2020), which could potentially result in greater salt sensitivity. The precise function of HvHKT1;5 in the salt‐including behavior of barley remains to be determined.

## Learning from halophytes

In the recent past, significant work has been carried out trying to understand the molecular mechanisms that plants use to cope with the saline condition. However, most of the work has been performed on glycophytic plants (salt‐sensitive plants) such as Arabidopsis, rice, tomato and maize. Compared to glycophytes, halophytes (salt‐resistant or salt‐tolerant plants) could provide the best model system for studying successful adaptation to salt stress (Shabala 2013). Concentrations of NaCl over 100 mM severely inhibit the growth of most glycophytes. On the contrary, halophytes usually complete their life cycles in soils considered highly saline, of at least 200 mM NaCl or even reaching 400 mM in the case of euhalophytes (Flowers and Colmer 2008). Halophytes exploit the same three specialized mechanisms as glycophytes to maintain a balanced K^+^:Na^+^ ratio in their cytosol: distribution of Na^+^ to various tissues, the export of Na^+^, and salt sequestration in vacuoles, all of them governed through transporters (Flowers and Colmer 2008, Flowers et al. 2010, Oh et al. 2010, Kronzucker and Britto 2011, Shabala 2013). The salt‐tolerance mechanisms in halophytes would be the same as those in glycophytes because they share common ancestry and evolution (Flowers et al. 2010). Evidence suggests that there are quantitative rather than qualitative differences between glycophytes and halophytes (Oh et al. 2010, Bartels and Dinakar 2013, Volkov 2015). This could occur due to an increased expression of genes related to the salt stress tolerance mechanism, or because halophyte proteins are more active than the corresponding glycophyte proteins, which indicate their better preparedness for harsh condition (Kumar et al. 2009, Das and Strasser 2013, Himabindu et al. 2016). The proteins belonging to HKT1‐type transporters and SOS pathway are a clear example of these differences. The halophytes studied to date possess HKT1 and SOS1 proteins that predominantly contribute to their halophytic nature (Oh et al. 2009, Taji et al. 2010, Dassanayake et al. 2011, Ali et al. 2012, Wu et al. 2012, Ali et al. 2018, Wang et al. 2020). However, their expression patterns are different in salt‐tolerant and sensitive varieties when subjected to NaCl stress. For instance, when the *HKT1* expression among halophytic and glycophytic species of *Cochlearia* was compared, much higher expression levels were found in the halophytic species than in the glycophytic, supporting the idea that an increase in *HKT* expression is crucial to high‐level salt tolerance (Nawaz et al. 2017). A comparison in the expression of genes in *A*. *thaliana* and *Eutrema salsuginea*, also revealed higher levels of *HKT1* in *E*. *salsuginea* compared to *A*. *thaliana* (Wu et al. 2012). In the same way, *EsHKT1;2* was strongly upregulated by salt stress while *EsHKT1;1* expression was much lower. Another interesting case occurs with *Salicornia dolichostachya*, a highly tolerant and salt accumulating halophyte. Compared with its glycophyte relative *Salicornia oleracea*, *S*. *dolichostachya* shows constitutively high levels of *SOS1* expression combined with suppression of *HKT1;1* in roots, suggesting a halotolerance strategy that strongly favors Na^+^ loading into the xylem sap for delivery to aerial parts (Katschnig et al. 2015). However, the expression of other *HKT1*‐like genes that might be present in *S*. *dolichostachya* was not explored.

As a close relative of Arabidopsis, *E*. *salsuginea* provides a model in which the halophytic nature of plants can be studied, drawing on the strengths of the glycophytic model and the exceptional ability to grow in seawater‐strength concentrations of NaCl (Inan et al. 2004, Gong et al. 2005, Amtmann 2009, Ali et al. 2013, Bartels and Dinakar 2013). Moreover, the genomes for *E*. *salsuginea* and the related *E*. *parvula* are sequenced, which opens the door to new insights about the genetic basis of abiotic stress (Dassanayake et al. 2011, Wu et al. 2012). In this regard, these two *Eutrema* species are good model plants for studying salinity stress tolerance mechanisms (Vera‐Estrella et al. 2005, Oh et al. 2009, 2010, Orsini et al. 2010, Volkov 2015).

**Fig. 2:**
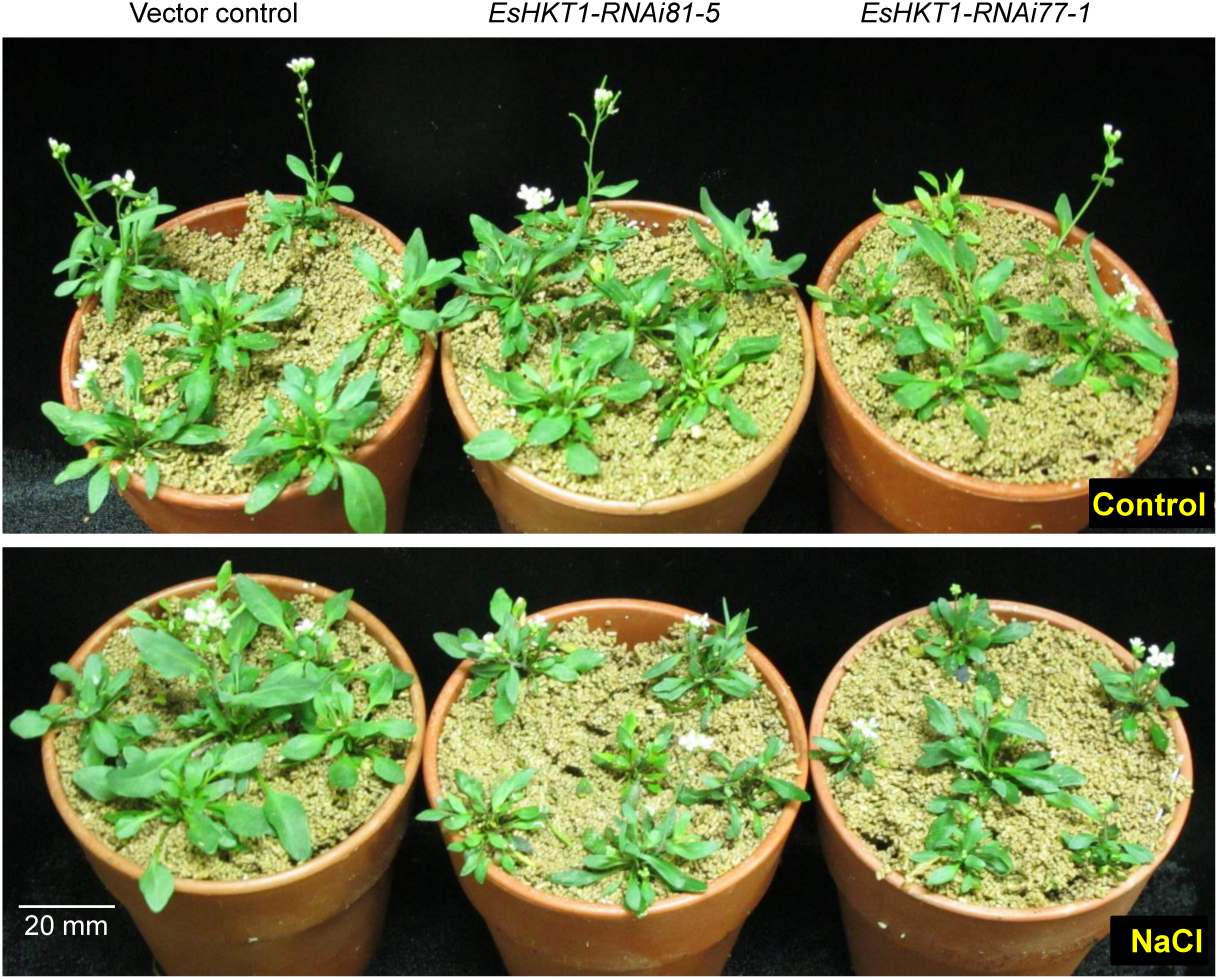
*EsHKT1‐RNAi* plants are sensitive to salt stress. *E*. *salsuginea* lines with knocked‐down expression of HKT1 (*EsHKT1‐RNAi*) are sensitive to high salinity. Four‐week‐old inert soil (porous soil) grown plants of the indicated genotypes without treatment (upper panel) or treated with 300 mM NaCl for 2 weeks (lower panel). Photographs were taken 2 weeks after salt stress. *EsHKT1‐RNAi* lines are more sensitive to salt stress as compared with vector control.

Previous studies indicate that salt tolerance in *E*. *salsugineum* does not arise due to complex morphological adaptations, but due to the robustness of the same mechanisms present in glycophytes (Bressan et al. 2001, Amtmann 2009). This adaptation can also occur through changes in gene regulation. Thus, *E*. *salsugineum* shows a higher level of expression in stress‐related genes than its orthologs in *A*. *thaliana*, suggesting that the transcriptional network may be regulated for better performance (Taji et al. 2004, Wong et al. 2006, Taji et al. 2010). This adaptive modification would favor, at least partially, the tightly controlled net absorption of Na^+^ and the efficient and selective absorption of K^+^ compared to Arabidopsis (Volkov et al. 2004, Ali et al. 2012, Oh et al. 2014, Ali et al. 2018). We may thus expect that a gradation in salt tolerance may be determined by some genes and proteins that differ not by type but by responsiveness to stress (Oh et al. 2009,Ali et al. 2012, Ali et al. 2018). In addition, comparison of the genome sequences of *E*. *salsuginea* and *E*. *parvula* to that of Arabidopsis might highlight informative differences in the number of isoform for stress‐related genes and their expressions such as *SOS*, *HKT*, and *NHX* (Oh et al. 2010, Ali et al. 2012, Wu et al. 2012, Oh et al. 2014, Ali et al. 2018). Recent studies reported the contribution of HKT1 isoforms to the halophytic character of *E*. *salsuginea* and *E*. *parvula* (Fig. 2; Ali et al. 2012, Ali et al. 2018), which we describe next.

## HKTs and halophytism

The critical role of HKT1 in Na^+^ retrieval from the xylem under salt stress is well established in the model plant *Arabidopsis thaliana* (Rus et al. 2001, Maser et al. 2002a, Sunarpi et al. 2005, Møller et al. 2009), and the crop plants tomato, rice and wheat (Ren et al. 2005, Munns et al. 2012, Almeida et al. 2014, Jaime‐Pérez et al. 2017). Considering the importance of Class I (HKT1) proteins in salt‐sensitive glycophytic plants, it is important to investigate their diversity and function in halophytic species. The *E*. *salsuginea* genome includes three *HKT1* genes in a tandem array (Wu et al. 2012). These genes encode for three proteins, of which EsHKT1;1 and EsHKT1;3 are Na^+^ transporters, while EsHKT1;2, is a Na^+^/K^+^ co‐transporter (Ali et al. 2012, Ali et al. 2016, Ali et al. 2018). Of the three homologs, only *EsHKT1;2*, is greatly induced following salt stress, highlighting its potential role in salt tolerance (Ali et al. 2012). By contrast, the *EsHKT1;1* transcript, like *AtHKT1*, is less activated under salt stress, whereas *EsHKT1;3* is expressed at low levels regardless of salt stress (Wu et al. 2012). *Eutrema parvula*, another Arabidopsis halophytic relative, contains two *HKT1* genes, encoding the Na^+^ transporter EpHKT1;1, and the Na^+^/K^+^ co‐transporter EpHKT1;2 (Dassanayake et al. 2011, Ali et al. 2018). Interestingly, *EpHKT1;2*, is upregulated upon salt stress, indicating again its likely contribution to the halophytic characteristics. On the other hand, upregulation of *EpHKT1;1* is much lower than that of *EpHKT1;2* (Ali et al. 2018). Finally, SsHKT1;1 from *Suaeda salsa*, a C3 halophyte, acts mainly as a K^+^ transporter and is involved in salt tolerance by maintaining cytosolic cation homeostasis, particularly K^+^ nutrition under salinity (Shao et al. 2014). These evidences indicate that homologs of HKT1‐type transporters, play a critical role to regulate Na^+^ toxicity in halophytes and are therefore principal components of halophytism.

Arabidopsis contains a single copy *HKT1* gene, encoding a Class I family member that shows a highly specific Na^+^ influx when expressed in *Xenopus laevis* oocytes and *Saccharomyces cerevisiae* (Uozumi et al. 2000). Based on their protein sequences, homologs of HKT1 from *E*. *salsuginea* and *E*. *parvula* contain a serine residue in the selectivity filter position of the first pore‐loop domain P_A_ (SGGG‐type) and therefore belong to Class I HKT1 transporters (Ali et al. 2012). However, isoforms from these halophytes, such as EsHK1;2 and EpHKT1;2, can take up both K^+^ and Na^+^, rather than only Na^+^, indicating that other important amino acids may control cation selectivity of these transporters apart from the S/G dichotomy in the selectivity filter (Ali et al. 2016). As we discussed earlier, HKT1 orthologs departing from the S/G rule in determining ion selectivity are found mostly in halophytes, suggesting an evolutionary advantage of this polymorphism in providing stress tolerance (Assaha et al. 2017).

**Fig. 3:**
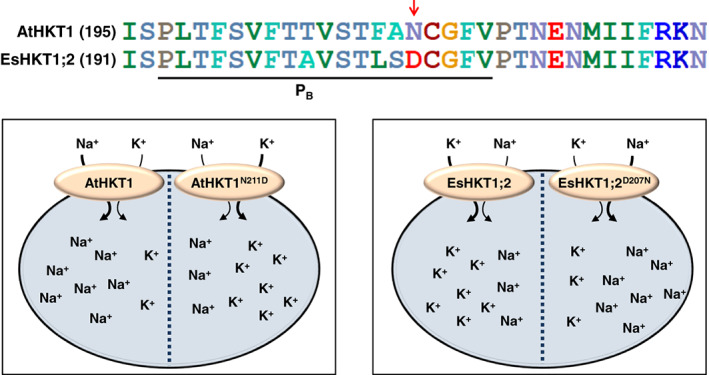
Cation selectivity of wild type AtHKT1 and EsHKT1;2 and their corresponding mutants. Protein sequences of AtHKT1 and EsHKT1;2 in the second pore‐loop domain (PB). Arrow indicates the conserved amino acid. The models explain the diverse cation selectivity of AtHKT1 (left) and EsHKT1;2 (right) and their mutants. AtHKT1 is a Na^+^‐specific transporter in plants, yeast, and oocytes with very low K^+^ selectivity in *E*. *coli*. By contrast, EsHKT1;2 is a high‐affinity K^+^ and Na^+^ co‐transporter. Substitution of Asn (N211) in AtHKT1 or Asp (D207) in EsHKT1;2 change their cation selectivity. AtHKT1^N211D^ functions like EsHKT1;2, whereas EsHKT1;2^D207N^ functions as AtHKT1, which highlights the importance of asp in the pore‐loop domain.

Alignment of amino acid sequences of three HKTs from *E*. *salsuginea*, two from *E*. *parvula* (now known as *Schrenkiella parvula*) and one from Arabidopsis (AtHKT1) with the well‐known high‐affinity K^+^ transporter of yeast, ScTRK1, provides clues about functional differences (Ali et al. 2012, 2016). Among them, EsHKT1;2 and EpHKT1;2 contain conserved aspartic acid residues (Asp207 and Asp205, respectively) in their second pore‐loop region (P_B_) and also in the adjacent transmembrane domain, M2_B_ (Asp238 and Asp236, respectively) (Ali et al. 2012). Interestingly, ScTRK1 possesses an Asp residue in the P_B_ (Ali et al. 2012). By contrast, the rest of the HKT1 proteins published to date contain an asparagine residue (Asn) at these positions (Ali et al. 2016). Surprisingly, SIHKT1;1 from tomato, a Class I HKT1 transporter, contains a serine residue (Ser265) at this position (Asins et al. 2013). These findings suggested that the presence of Asp in the P_A_ region of halophytic HKT1 transporters could be responsible for K^+^ selectively rather than Na^+^. Indeed, the substitution of Asp207 or Asp205 by Asn in EsHKT1;2 and EpHKT1;2, respectively, abolished K^+^ uptake, generating a canonical Class I Na^+^‐selective transporter (Ali et al. 2016, 2018). In addition, plants expressing *EsHKT1;2* or *EpHKT1;2* in an *athkt1* knockout background tolerate salt more effectively than those expressing *AtHKT1*. However, unlike *EsHKT1;2*, plants expressing *EsHKT1;2* ^*D207N*^ become sensitive to high salinity exactly like those expressing *AtHKT1* (Ali and Yun 2016). Conversely, plants expressing *AtHKT1* ^*N211D*^, where Asn211 is replaced by Asp211 show significant salt tolerance and gain the ability to take up K^+^ as well. Thus, by changing the asparagine residue in the P_B_ region of AtHKT1 to aspartic acid, the transporter comes to resemble EsHKT1;2, with a high affinity for K^+^ transport resulting in salt tolerance (Ali et al. 2016; Fig. 3).

Recently, the mutagenesis of VisHKT1;1 (for *Vitis interspecific*) and TaHKT1;5‐D from bread wheat (*Triticum aestivum*), established that charged amino acid residues located in the eighth transmembrane domain, M2_D_, converted these transporters into inward rectifiers of Na^+^ (Henderson et al. 2018). Thus, it seems clear that small changes in HKT proteins can improve salt tolerance. These results also suggest that through genome duplication and SNPs, halophytic HKTs gained the ability to take up K^+^ contributing to the halophytic nature of the species.

## Conclusions and future perspectives

SOS1 and HKT1‐like proteins are among the most important transporters that plants, glycophytes and halophytes alike, use to deal with high salinity. SOS1 exports Na^+^ out of the root and facilitates the loading into the xylem, thereby protecting the root from salt‐induced damages. By contrast, HKT1‐like proteins unload Na^+^ from xylem and help sequestering ions into xylem parenchyma cells to protect photosynthetic tissues. These two transporter systems function antagonistically in a refined way to spare the plant from Na^+^ toxicity, yet how their activities are co‐regulated and fine‐tuned to avoid a futile cycle of Na^+^ loading and unloading remains unknown.

Functional characterization of the SOS pathway and HKT1‐type transporters in *A*. *thaliana* and *E*. *salsuginea* hint that these plant species use similar mechanisms to tolerate salinity. The question then is why is Arabidopsis salt‐sensitive whereas its halophytic counterpart shows extreme tolerance toward salinity? Several factors could explain how *E*. *salsuginea* is different from Arabidopsis; for instance, the genes encoding the Na^+^/H^+^ antiporter (*SOS1*) and Na^+^/K^+^ transporter (*HKT1*) are more strongly induced in *E*. *salsuginea* compared to Arabidopsis (Oh et al. 2009, Wu et al. 2012), suggesting greater preparedness and responsiveness in the halophytic plant. Gene duplication of HKT1 homologs and neo‐functionalization in *E*. *salsuginea* is another possible explanation (Dassanayake et al. 2011, Wu et al. 2012). These differences point to the co‐evolution of the two ion transport systems playing a critical role in shaping the halophytic lifestyle of *E*. *salsuginea*. Furthermore, current knowledge suggests that halophytes possess highly developed and upgraded mechanisms that are presumably related to those present in glycophytes (Oh et al. 2014). Thus, the study of halophytic plants such as *E*. *salsuginea* and *S*. *parvula* that are close relatives to Arabidopsis may advance our knowledge of how plants achieve salt tolerance by using a similar repertoire of molecular resources. Combined with functional studies in Arabidopsis, analysis of homologous ion transporter, and their divergence in *E*. *salsuginea* may help us to understand how multiple ion transporters that contribute to ion tolerance have been acquired evolutionarily, permitting survival in environments with extreme levels of sodium ions. Gaining knowledge of the responses and strategies used by halophytes under saline conditions could allow their application to crop plants in the future.

## Author contributions

All authors designed the work, collected information from literature and wrote the paper.

## Data Availability

Data sharing is not applicable to this article as no new data were created or analyzed in this study.

## References

[ppl13166-bib-0001] Ali A , Cheol Park H , Aman R , Ali Z , Yun DJ (2013) Role of HKT1 in *Thellungiella salsuginea*, a model extremophile plant. Plant Signal Behav 8(8), e25196 23759555 10.4161/psb.25196 PMC3999061

[ppl13166-bib-0002] Ali A , Khan IU , Jan M , Khan HA , Hussain S , Nisar M , Chung WS , Yun DJ (2018) The high‐affinity potassium transporter EpHKT1;2 from the extremophile *Eutrema parvula* mediates salt tolerance. Front Plant Sci 9: 1108 30105045 10.3389/fpls.2018.01108 PMC6077265

[ppl13166-bib-0003] Ali A , Raddatz N , Aman R , Kim S , Park HC , Jan M , Baek D , Khan IU , Oh DH , Lee SY , Bressan RA , Lee KW , Maggio A , Pardo JM , Bohnert HJ , Yun DJ (2016) A single amino‐acid substitution in the sodium transporter HKT1 associated with plant salt tolerance. Plant Physiol 171: 2112–2126 27208305 10.1104/pp.16.00569 PMC4936583

[ppl13166-bib-0004] Ali A , Yun DJ (2016) Differential selection of sodium and potassium ions by TsHKT1;2. Plant Signal Behav 11: e1206169 27380309 10.1080/15592324.2016.1206169 PMC5022409

[ppl13166-bib-0005] Ali Z , Park HC , Ali A , Oh DH , Aman R , Kropornicka A , Hong H , Choi W , Chung WS , Kim WY , Bressan RA , Bohnert HJ , Lee SY , Yun DJ (2012) TsHKT1;2, a HKT1 homolog from the extremophile *Arabidopsis* relative *Thellungiella salsuginea*, shows K(+) specificity in the presence of NaCl. Plant Physiol 158: 1463–1474 22238420 10.1104/pp.111.193110 PMC3291249

[ppl13166-bib-0006] Almeida P , de Boer GJ , de Boer AH (2014) Differences in shoot Na^+^ accumulation between two tomato species are due to differences in ion affinity of HKT1;2. J Plant Physiol 171: 438–447 24594396 10.1016/j.jplph.2013.12.001

[ppl13166-bib-0007] Amtmann A (2009) Learning from evolution: *Thellungiella* generates new knowledge on essential and critical components of abiotic stress tolerance in plants. Mol Plant 2: 3–12 19529830 10.1093/mp/ssn094 PMC2639741

[ppl13166-bib-0008] Asins MJ , Villalta I , Aly MM , Olias R , Alvarez DEMP , Huertas R , Li J , Jaime‐Perez N , Haro R , Raga V , Carbonell EA , Belver A (2013) Two closely linked tomato HKT coding genes are positional candidates for the major tomato QTL involved in Na+ /K+ homeostasis. Plant Cell Environ 36: 1171–1191 23216099 10.1111/pce.12051

[ppl13166-bib-0009] Assaha DVM , Ueda A , Saneoka H , Al‐Yahyai R , Yaish MW (2017) The role of Na^+^ and K^+^ transporters in salt stress adaptation in Glycophytes. Front Physiol 8: 509 28769821 10.3389/fphys.2017.00509 PMC5513949

[ppl13166-bib-0010] Barajas‐Lopez JD , Moreno JR , Gamez‐Arjona FM , Pardo JM , Punkkinen M , Zhu JK , Quintero FJ , Fujii H (2018) Upstream kinases of plant SnRKs are involved in salt stress tolerance. Plant J 93: 107–118 29094495 10.1111/tpj.13761 PMC5814739

[ppl13166-bib-0011] Bartels D , Dinakar C (2013) Balancing salinity stress responses in halophytes and non‐halophytes: a comparison between *Thellungiella* and *Arabidopsis thaliana* . J Functional Plant Biology 40: 819–831 10.1071/FP12299 32481153

[ppl13166-bib-0012] Benito B , Haro R , Amtmann A , Cuin TA , Dreyer I (2014) The twins K+ and Na+ in plants. J Plant Physiol 171: 723–731 24810769 10.1016/j.jplph.2013.10.014

[ppl13166-bib-0013] Bressan RA , Zhang C , Zhang H , Hasegawa PM , Bohnert HJ , Zhu JK (2001) Learning from the *Arabidopsis* experience. The next gene search paradigm. Plant Physiol 127: 1354–1360 11743073 PMC1540162

[ppl13166-bib-0014] Cao Y , Jin X , Huang H , Derebe MG , Levin EJ , Kabaleeswaran V , Pan Y , Punta M , Love J , Weng J (2011) Crystal structure of a potassium ion transporter, TrkH. Nature 471: 336–340 21317882 10.1038/nature09731 PMC3077569

[ppl13166-bib-0015] Cao Y , Liang X , Yin P , Zhang M , Jiang C (2019) A domestication‐associated reduction in K(+) ‐preferring HKT transporter activity underlies maize shoot K(+) accumulation and salt tolerance. New Phytol 222: 301–317 30461018 10.1111/nph.15605

[ppl13166-bib-0016] Chaves MM , Flexas J , Pinheiro C (2009) Photosynthesis under drought and salt stress: regulation mechanisms from whole plant to cell. Ann Bot 103: 551–560 18662937 10.1093/aob/mcn125 PMC2707345

[ppl13166-bib-0017] Cotsaftis O , Plett D , Shirley N , Tester M , Hrmova M (2012) A two‐staged model of Na(+) exclusion in rice explained by 3D modeling of HKT transporters and alternative splicing. PLoS One 7: e39865 22808069 10.1371/journal.pone.0039865 PMC3394774

[ppl13166-bib-0018] Das AB , Strasser RJ (2013) Salinity‐induced genes and molecular basis of salt‐tolerant strategies in mangroves. In GR Rout and AB Das (eds.). (Springer), Molecular Stress Physiology of Plants, pp 53–86

[ppl13166-bib-0019] Dassanayake M , Oh DH , Haas JS , Hernandez A , Hong H , Ali S , Yun DJ , Bressan RA , Zhu JK , Bohnert HJ , Cheeseman JM (2011) The genome of the extremophile crucifer *Thellungiella parvula* . Nat Genet 43: 913–918 21822265 10.1038/ng.889 PMC3586812

[ppl13166-bib-0020] Davenport RJ , Munoz‐Mayor A , Jha D , Essah PA , Rus A , Tester M (2007) The Na+ transporter AtHKT1;1 controls retrieval of Na+ from the xylem in *Arabidopsis* . Plant Cell Environ 30: 497–507 17324235 10.1111/j.1365-3040.2007.01637.x

[ppl13166-bib-0021] Demidchik V (2014) Mechanisms and physiological roles of K^+^ efflux from root cells. J Plant Physiol 171: 696–707 24685330 10.1016/j.jplph.2014.01.015

[ppl13166-bib-0022] El Mahi H , Perez‐Hormaeche J , De Luca A , Villalta I , Espartero J , Gamez‐Arjona F , Fernandez JL , Bundo M , Mendoza I , Mieulet D , Lalanne E , Lee SY , Yun DJ , Guiderdoni E , Aguilar M , Leidi EO , Pardo JM , Quintero FJ (2019) A critical role of sodium flux via the plasma membrane Na(+)/H(+) exchanger SOS1 in the salt tolerance of rice. Plant Physiol 180: 1046–1065 30992336 10.1104/pp.19.00324 PMC6548274

[ppl13166-bib-0023] Fairbairn DJ , Liu W , Schachtman DP , Gomez‐Gallego S , Day SR , Teasdale RD (2000) Characterisation of two distinct HKT1‐like potassium transporters from *Eucalyptus camaldulensis* . Plant Mol Biol 43: 515–525 11052203 10.1023/a:1006496402463

[ppl13166-bib-0024] Flowers TJ , Colmer TD (2008) Salinity tolerance in halophytes. New Phytol 179: 945–963 18565144 10.1111/j.1469-8137.2008.02531.x

[ppl13166-bib-0025] Flowers TJ , Galal HK , Bromham L (2010) Evolution of halophytes: multiple origins of salt tolerance in land plants. Functional Plant Biol 37: 604–612

[ppl13166-bib-0026] Fuglsang AT , Guo Y , Cuin TA , Qiu Q , Song C , Kristiansen KA , Bych K , Schulz A , Shabala S , Schumaker KS , Palmgren MG , Zhu J‐K (2007) *Arabidopsis* protein kinase PKS5 inhibits the plasma membrane H+‐ATPase by preventing interaction with 14‐3‐3 protein. Plant Cell 19: 1617–1634 17483306 10.1105/tpc.105.035626 PMC1913743

[ppl13166-bib-0027] Garciadeblas B , Senn ME , Banuelos MA , Rodriguez‐Navarro A (2003) Sodium transport and HKT transporters: the rice model. Plant J 34: 788–801 12795699 10.1046/j.1365-313x.2003.01764.x

[ppl13166-bib-0028] Golldack D , Su H , Quigley F , Kamasani UR , Munoz‐Garay C , Balderas E , Popova OV , Bennett J , Bohnert HJ , Pantoja O (2002) Characterization of a HKT‐type transporter in rice as a general alkali cation transporter. Plant J 31: 529–542 12182709 10.1046/j.1365-313x.2002.01374.x

[ppl13166-bib-0029] Gong Q , Li P , Ma S , Indu Rupassara S , Bohnert HJ (2005) Salinity stress adaptation competence in the extremophile *Thellungiella halophila* in comparison with its relative *Arabidopsis thaliana* . Plant J 44: 826–839 16297073 10.1111/j.1365-313X.2005.02587.x

[ppl13166-bib-0030] Hamamoto S , Horie T , Hauser F , Deinlein U , Schroeder JI , Uozumi N (2015) HKT transporters mediate salt stress resistance in plants: from structure and function to the field. Curr Opin Biotechnol 32: 113–120 25528276 10.1016/j.copbio.2014.11.025

[ppl13166-bib-0031] Hanin M , Ebel C , Ngom M , Laplaze L , Masmoudi K (2016) New insights on plant salt tolerance mechanisms and their potential use for breeding. Front Plant Sci 7: 1787 27965692 10.3389/fpls.2016.01787 PMC5126725

[ppl13166-bib-0032] Hauser F , Horie T (2010) A conserved primary salt tolerance mechanism mediated by HKT transporters: a mechanism for sodium exclusion and maintenance of high K+/Na+ ratio in leaves during salinity stress. Plant Cell Environ 33: 552–565 19895406 10.1111/j.1365-3040.2009.02056.x

[ppl13166-bib-0033] Henderson SW , Dunlevy JD , Wu Y , Blackmore DH , Walker RR , Edwards EJ , Gilliham M , Walker AR (2018) Functional differences in transport properties of natural HKT1;1 variants influence shoot Na(+) exclusion in grapevine rootstocks. New Phytol 217: 1113–1127 29160564 10.1111/nph.14888

[ppl13166-bib-0034] Himabindu Y , Chakradhar T , Reddy MC , Kanygin A , Redding KE , Chandrasekhar T (2016) Salt‐tolerant genes from halophytes are potential key players of salt tolerance in glycophytes. J Environ Exp Botany 124: 39–63

[ppl13166-bib-0035] Hmidi D , Messedi D , Corratgi‐Faillie C , Marhuenda TO , Fizames CC , Zorrig W , Abdelly C , Sentenac H , Vi Ry AN (2019) Investigation of Na(+) and K(+) transport in halophytes: functional analysis of the HmHKT2;1 transporter from *Hordeum maritimum* and expression under saline conditions. Plant Cell Physiol 60: 2423–2435 31292634 10.1093/pcp/pcz136

[ppl13166-bib-0036] Horie T , Costa A , Kim TH , Han MJ , Horie R , Leung HY , Miyao A , Hirochika H , An G , Schroeder JI (2007) Rice OsHKT2;1 transporter mediates large Na(+) influx component into K(+)‐starved roots for growth. EMBO J 26: 3003–3014 17541409 10.1038/sj.emboj.7601732 PMC1894770

[ppl13166-bib-0037] Horie T , Hauser F , Schroeder JI (2009) HKT transporter‐mediated salinity resistance mechanisms in *Arabidopsis* and monocot crop plants. Trends Plant Sci 14: 660–668 19783197 10.1016/j.tplants.2009.08.009 PMC2787891

[ppl13166-bib-0038] Horie T , Yoshida K , Nakayama H , Yamada K , Oiki S , Shinmyo A (2001) Two types of HKT transporters with different properties of Na(+) and K(+) transport in *Oryza sativa* . Plant J 27: 129–138 11489190 10.1046/j.1365-313x.2001.01077.x

[ppl13166-bib-0039] Huang L , Kuang L , Wu L , Shen Q , Han Y , Jiang L , Wu D , Zhang G (2020) The HKT transporter HvHKT1;5 negatively regulates salt tolerance. Plant Physiol 182: 584–596 31690708 10.1104/pp.19.00882 PMC6945855

[ppl13166-bib-0040] Inan G , Zhang Q , Li PH , Wang ZL , Cao ZY , Zhang H , Zhang CQ , Quist TM , Goodwin SM , Zhu JH , Shi HH , Damsz B , Charbaji T , Gong QQ , Ma SS , Fredricksen M , Galbraith DW , Jenks MA , Rhodes D , Hasegawa PM , Bohnert HJ , Joly RJ , Bressan RA , Zhu JK (2004) Salt cress. A halophyte and cryophyte *Arabidopsis* relative model system and its applicability to molecular genetic analyses of growth and development of extremophiles. Plant Physiol 135: 1718–1737 15247369 10.1104/pp.104.041723 PMC519085

[ppl13166-bib-0041] Isayenkov S , Maathuis FJM (2019) Plant salinity stress; many unanswered questions remain. Front Plant Sci 10: 1–11 30828339 10.3389/fpls.2019.00080 PMC6384275

[ppl13166-bib-0042] Jabnoune M , Espeout S , Mieulet D , Fizames C , Verdeil JL , Conejero G , Rodriguez‐Navarro A , Sentenac H , Guiderdoni E , Abdelly C , Very AA (2009) Diversity in expression patterns and functional properties in the rice HKT transporter family. Plant Physiol 150: 1955–1971 19482918 10.1104/pp.109.138008 PMC2719131

[ppl13166-bib-0043] Jaime‐Pérez N , Pineda B , García‐Sogo B , Atares A , Athman A , Byrt CS , Olías R , Asins MJ , Gilliham M , Moreno V , Belver A (2017) The sodium transporter encoded by the *HKT1;2* gene modulates sodium/potassium homeostasis in tomato shoots under salinity. Plant Cell Environ 40: 658–671 27987209 10.1111/pce.12883

[ppl13166-bib-0044] James RA , Blake C , Byrt CS , Munns R (2011) Major genes for Na+ exclusion, Nax1 and Nax2 (wheat HKT1;4 and HKT1;5), decrease Na+ accumulation in bread wheat leaves under saline and waterlogged conditions. J Exp Bot 62: 2939–2947 21357768 10.1093/jxb/err003

[ppl13166-bib-0045] Ji H , Pardo JM , Batelli G , Van Oosten MJ , Bressan RA , Li X (2013) The salt overly sensitive (SOS) pathway: established and emerging roles. Mol Plant 6: 275–286 23355543 10.1093/mp/sst017

[ppl13166-bib-0046] Kader MA , Seidel T , Golldack D , Lindberg S (2006) Expressions of OsHKT1, OsHKT2, and OsVHA are differentially regulated under NaCl stress in salt‐sensitive and salt‐tolerant rice (*Oryza sativa* L.) cultivars. J Exp Bot 57: 4257–4268 17088362 10.1093/jxb/erl199

[ppl13166-bib-0047] Katschnig D , Bliek T , Rozema J , Schat H (2015) Constitutive high‐level SOS1 expression and absence of HKT1;1 expression in the salt‐accumulating halophyte *Salicornia dolichostachya* . Plant Sci 234: 144–154 25804817 10.1016/j.plantsci.2015.02.011

[ppl13166-bib-0048] Kim BG , Waadt R , Cheong YH , Pandey GK , Dominguez‐Solis JR , Schultke S , Lee SC , Kudla J , Luan S (2007) The calcium sensor CBL10 mediates salt tolerance by regulating ion homeostasis in *Arabidopsis* . Plant J 52: 473–484 17825054 10.1111/j.1365-313X.2007.03249.x

[ppl13166-bib-0049] Kim WY , Ali Z , Park HJ , Park SJ , Cha JY , Perez‐Hormaeche J , Quintero FJ , Shin G , Kim MR , Qiang Z , Ning L , Park HC , Lee SY , Bressan RA , Pardo JM , Bohnert HJ , Yun DJ (2013) Release of SOS2 kinase from sequestration with GIGANTEA determines salt tolerance in *Arabidopsis* . Nat Commun 4: 1352 23322040 10.1038/ncomms2357

[ppl13166-bib-0050] Kronzucker HJ , Britto DT (2011) Sodium transport in plants: a critical review. New Phytol 189: 54–81 21118256 10.1111/j.1469-8137.2010.03540.x

[ppl13166-bib-0051] Kumar G , Purty RS , Singla‐Pareek SL , Pareek A (2009) Maintenance of stress related transcripts in tolerant cultivar at a level higher than sensitive one appears to be a conserved salinity response among plants. Plant Signal Behav 4: 431–434 19816099 10.4161/psb.4.5.8298 PMC2676757

[ppl13166-bib-0053] Li K , Wang Y , Han C , Zhang W , Jia H , Li XT (2007) GA signaling and CO/FT regulatory module mediate salt‐induced late flowering in *Arabidopsis thaliana* . Plant Growth Regul 53: 195–206

[ppl13166-bib-0054] Luo M , Zhao Y , Zhang R , Xing J , Duan M , Li J , Wang N , Wang W , Zhang S , Chen Z , Zhang H , Shi Z , Song W , Zhao J (2017) Mapping of a major QTL for salt tolerance of mature field‐grown maize plants based on SNP markers. BMC Plant Biol 17: 140 28806927 10.1186/s12870-017-1090-7 PMC5556339

[ppl13166-bib-0055] Maathuis FJ (2014) Sodium in plants: perception, signalling, and regulation of sodium fluxes. J Exp Bot 65: 849–858 24151301 10.1093/jxb/ert326

[ppl13166-bib-0056] Maser P , Eckelman B , Vaidyanathan R , Horie T , Fairbairn DJ , Kubo M , Yamagami M , Yamaguchi K , Nishimura M , Uozumi N , Robertson W , Sussman MR , Schroeder JI (2002a) Altered shoot/root Na(+) distribution and bifurcating salt sensitivity in *Arabidopsis* by genetic disruption of the Na(+) transporter AtHKT1. FEBS Lett 531: 157–161 12417304 10.1016/s0014-5793(02)03488-9

[ppl13166-bib-0057] Maser P , Hosoo Y , Goshima S , Horie T , Eckelman B , Yamada K , Yoshida K , Bakker EP , Shinmyo A , Oiki S , Schroeder JI , Uozumi N (2002b) Glycine residues in potassium channel‐like selectivity filters determine potassium selectivity in four‐loop‐per‐subunit HKT transporters from plants. Proc Natl Acad Sci U S A 99: 6428–6433 11959905 10.1073/pnas.082123799 PMC122965

[ppl13166-bib-0058] Mian A , Oomen RJ , Isayenkov S , Sentenac H , Maathuis FJ , Very AA (2011) Over‐expression of an Na(+)‐and K(+)‐permeable HKT transporter in barley improves salt tolerance. Plant J 68: 468–479 21749504 10.1111/j.1365-313X.2011.04701.x

[ppl13166-bib-0059] Møller IS , Gilliham M , Jha D , Mayo GM , Roy SJ , Coates JC , Haseloff J , Tester M (2009) Shoot Na^+^ exclusion and increased salinity tolerance engineered by cell type‐specific alteration of Na^+^ transport in *Arabidopsis* . Plant Cell 217: 2163–2178 10.1105/tpc.108.064568 PMC2729596 19584143

[ppl13166-bib-0060] Munns R (2002) Comparative physiology of salt and water stress. Plant Cell and Environ 25: 239–250 10.1046/j.0016-8025.2001.00808.x 11841667

[ppl13166-bib-0061] Munns R , James RA , Xu B , Athman A , Conn SJ , Jordans C , Byrt CS , Hare RA , Tyerman SD , Tester M , Plett D , Gilliham M (2012) Wheat grain yield on saline soils is improved by an ancestral Na(+) transporter gene. Nat Biotechnol 30: 360–364 22407351 10.1038/nbt.2120

[ppl13166-bib-0062] Nawaz I , Iqbal M , Bliek M , Schat H (2017) Salt and heavy metal tolerance and expression levels of candidate tolerance genes among four extremophile Cochlearia species with contrasting habitat preferences. Sci Total Environ 584‐585: 731–741 10.1016/j.scitotenv.2017.01.111 28129909

[ppl13166-bib-0063] Nieves‐Cordones M , Martinez V , Benito B , Rubio F (2016) Comparison between Arabidopsis and rice for main pathways of K(+) and Na(+) uptake by roots. Front Plant Sci 7: 992 27458473 10.3389/fpls.2016.00992 PMC4932104

[ppl13166-bib-0064] Oh DH , Dassanayake M , Haas JS , Kropornika A , Wright C , d'Urzo MP , Hong H , Ali S , Hernandez A , Lambert GM , Inan G , Galbraith DW , Bressan RA , Yun DJ , Zhu JK , Cheeseman JM , Bohnert HJ (2010) Genome structures and halophyte‐specific gene expression of the extremophile *Thellungiella parvula* in comparison with *Thellungiella salsuginea* (*Thellungiella halophila*) and Arabidopsis. Plant Physiol 154: 1040–1052 20833729 10.1104/pp.110.163923 PMC2971586

[ppl13166-bib-0065] Oh DH , Hong H , Lee SY , Yun DJ , Bohnert HJ , Dassanayake M (2014) Genome structures and transcriptomes signify niche adaptation for the multiple‐ion‐tolerant extremophyte Schrenkiella parvula. Plant Physiol 164: 2123–2138 24563282 10.1104/pp.113.233551 PMC3982767

[ppl13166-bib-0066] Oh DH , Leidi E , Zhang Q , Hwang SM , Li Y , Quintero FJ , Jiang X , D'Urzo MP , Lee SY , Zhao Y , Bahk JD , Bressan RA , Yun DJ , Pardo JM , Bohnert HJ (2009) Loss of halophytism by interference with SOS1 expression. Plant Physiol 151: 210–222 19571313 10.1104/pp.109.137802 PMC2735974

[ppl13166-bib-0067] Olias R , Eljakaoui Z , Li J , De Morales PA , Marin‐Manzano MC , Pardo JM , Belver A (2009) The plasma membrane Na+/H+ antiporter SOS1 is essential for salt tolerance in tomato and affects the partitioning of Na+ between plant organs. Plant Cell Environ 32: 904–916 19302170 10.1111/j.1365-3040.2009.01971.x

[ppl13166-bib-0068] Oomen RJ , Benito B , Sentenac H , Rodriguez‐Navarro A , Talon M , Very AA , Domingo C (2012) HKT2;2/1, a K(+)‐permeable transporter identified in a salt‐tolerant rice cultivar through surveys of natural genetic polymorphism. Plant J 71: 750–762 22530609 10.1111/j.1365-313X.2012.05031.x

[ppl13166-bib-0069] Orsini F , D'Urzo MP , Inan G , Serra S , Oh DH , Mickelbart MV , Consiglio F , Li X , Jeong JC , Yun DJ , Bohnert HJ , Bressan RA , Maggio A (2010) A comparative study of salt tolerance parameters in 11 wild relatives of *Arabidopsis thaliana* . J Exp Bot 61: 3787–3798 20595237 10.1093/jxb/erq188 PMC2921208

[ppl13166-bib-0070] Pires IS , Negrao S , Pentony MM , Abreu IA , Oliveira MM , Purugganan MD (2013) Different evolutionary histories of two cation/proton exchanger gene families in plants. BMC Plant Biol 13: 97 23822194 10.1186/1471-2229-13-97 PMC3726471

[ppl13166-bib-0071] Platten JD , Cotsaftis O , Berthomieu P , Bohnert H , Davenport RJ , Fairbairn DJ , Horie T , Leigh RA , Lin HX , Luan S , Maser P , Pantoja O , Rodriguez‐Navarro A , Schachtman DP , Schroeder JI , Sentenac H , Uozumi N , Very AA , Zhu JK , Dennis ES , Tester M (2006) Nomenclature for HKT transporters, key determinants of plant salinity tolerance. Trends Plant Sci 11: 372–374 16809061 10.1016/j.tplants.2006.06.001

[ppl13166-bib-0072] Qi Z , Spalding EP (2004) Protection of plasma membrane K+ transport by the salt overly sensitive1 Na^+^‐H^+^ antiporter during salinity stress. Plant Physiol 136: 2548–2555 15347782 10.1104/pp.104.049213 PMC523321

[ppl13166-bib-0073] Qiu QS , Guo Y , Dietrich MA , Schumaker KS , Zhu JK (2002) Regulation of SOS1, a plasma membrane Na^+^/H^+^ exchanger in *Arabidopsis thaliana*, by SOS2 and SOS3. Proceedings of the National Academy of Sciences of the United States of America 99: 8436–8441 12034882 10.1073/pnas.122224699 PMC123085

[ppl13166-bib-0074] Quan R , Lin H , Mendoza I , Zhang Y , Cao W , Yang Y , Shang M , Chen S , Pardo JM , Guo Y (2007) SCABP8/CBL10, a putative calcium sensor, interacts with the protein kinase SOS2 to protect Arabidopsis shoots from salt stress. Plant Cell 19: 1415–1431 17449811 10.1105/tpc.106.042291 PMC1913747

[ppl13166-bib-0075] Quintero FJ , Martinez‐Atienza J , Villalta I , Jiang X , Kim WY , Ali Z , Fujii H , Mendoza I , Yun DJ , Zhu JK , Pardo JM (2011) Activation of the plasma membrane Na/H antiporter salt‐overly‐sensitive 1 (SOS1) by phosphorylation of an auto‐inhibitory C‐terminal domain. Proc Natl Acad Sci U S A 108: 2611–2616 21262798 10.1073/pnas.1018921108 PMC3038701

[ppl13166-bib-0076] Quintero FJ , Ohta M , Shi H , Zhu JK , Pardo JM (2002) Reconstitution in yeast of the Arabidopsis SOS signaling pathway for Na+ homeostasis. Proc Natl Acad Sci U S A 99: 9061–9066 12070350 10.1073/pnas.132092099 PMC124423

[ppl13166-bib-0077] Raddatz N , Morales de Los Rios L , Lindahl M , Quintero FJ , Pardo JM (2020) Coordinated transport of nitrate, potassium, and sodium. Front Plant Sci 11: 247 32211003 10.3389/fpls.2020.00247 PMC7067972

[ppl13166-bib-0078] Ren ZH , Gao JP , Li LG , Cai XL , Huang W , Chao DY , Zhu MZ , Lin HX (2005) A rice quantitative trait locus for salt tolerance encodes a sodium transporter. Nat Genet 37: 1141–1146 16155566 10.1038/ng1643

[ppl13166-bib-0079] Riedelsberger J , Vergara‐Jaque A , Piñeros M , Dreyer I , González W (2018) Extracellular cation binding pocket is essential for ion conduction of OsHKT2; 2. bioRxiv: 471003. Available at https://www.biorxiv.org/content/10.1101/471003v1.abstract (accessed November 16, 2018).

[ppl13166-bib-0080] Rivandi J , Miyazaki J , Hrmova M , Pallotta M , Tester M , Collins NC (2011) A SOS3 homologue maps to HvNax4, a barley locus controlling an environmentally sensitive Na+ exclusion trait. J Exp Bot 62: 1201–1216 21047983 10.1093/jxb/erq346 PMC3022402

[ppl13166-bib-0081] Rubio F , Nieves‐Cordones M , Horie T , Shabala S (2020) Doing 'business as usual' comes with a cost: evaluating energy cost of maintaining plant intracellular K^+^ homeostasis under saline conditions. New Phytol 225: 1097–1104 30993727 10.1111/nph.15852

[ppl13166-bib-0082] Rus A , Yokoi S , Sharkhuu A , Reddy M , Lee BH , Matsumoto TK , Koiwa H , Zhu JK , Bressan RA , Hasegawa PM (2001) AtHKT1 is a salt tolerance determinant that controls Na(+) entry into plant roots. Proc Natl Acad Sci U S A 98: 14150–14155 11698666 10.1073/pnas.241501798 PMC61183

[ppl13166-bib-0083] Schachtman DP , Schroeder JI (1994) Structure and transport mechanism of a high‐affinity potassium uptake transporter from higher plants. Nature 370: 655–658 8065452 10.1038/370655a0

[ppl13166-bib-0084] Shabala S (2013) Learning from halophytes: physiological basis and strategies to improve abiotic stress tolerance in crops. Ann Bot 112: 1209–1221 24085482 10.1093/aob/mct205 PMC3806534

[ppl13166-bib-0085] Shabala S , Pottosin I (2014) Regulation of potassium transport in plants under hostile conditions: implications for abiotic and biotic stress tolerance. Physiol Plant 151: 257–279 24506225 10.1111/ppl.12165

[ppl13166-bib-0086] Shao Q , Han N , Ding T , Zhou F , Wang B (2014) SsHKT1;1 is a potassium transporter of the C3 halophyte Suaeda salsa that is involved in salt tolerance J. Funct Plant Biol 41: 790–802 32481033 10.1071/FP13265

[ppl13166-bib-0087] Shi H , Quintero FJ , Pardo JM , Zhu JK (2002) The putative plasma membrane Na(+)/H(+) antiporter SOS1 controls long‐distance Na(+) transport in plants. Plant Cell 14: 465–477 11884687 10.1105/tpc.010371 PMC152925

[ppl13166-bib-0088] Silveira JAG , Carvalho FEL (2016) Proteomics, photosynthesis and salt resistance in crops: An integrative view. J Proteomics 143: 24–35 26957143 10.1016/j.jprot.2016.03.013

[ppl13166-bib-0089] Su H , Balderas E , Vera‐Estrella R , Golldack D , Quigley F , Zhao C , Pantoja O , Bohnert HJ (2003) Expression of the cation transporter McHKT1 in a halophyte. Plant Mol Biol 52: 967–980 14558658 10.1023/a:1025445612244

[ppl13166-bib-0090] Sunarpi HT , Motoda J , Kubo M , Yang H , Yoda K , Horie R , Chan WY , Leung HY , Hattori K , Konomi M , Osumi M , Yamagami M , Schroeder JI , Uozumi N (2005) Enhanced salt tolerance mediated by AtHKT1 transporter‐induced Na(+) unloading from xylem vessels to xylem parenchyma cells. Plant J 44: 928–938 16359386 10.1111/j.1365-313X.2005.02595.x

[ppl13166-bib-0091] Taji T , Komatsu K , Katori T , Kawasaki Y , Sakata Y , Tanaka S , Kobayashi M , Toyoda A , Seki M , Shinozaki K (2010) Comparative genomic analysis of 1047 completely sequenced cDNAs from an Arabidopsis‐related model halophyte, Thellungiella halophila. BMC Plant Biol 10: 261 21106055 10.1186/1471-2229-10-261 PMC3017837

[ppl13166-bib-0092] Taji T , Seki M , Satou M , Sakurai T , Kobayashi M , Ishiyama K , Narusaka Y , Narusaka M , Zhu JK , Shinozaki K (2004) Comparative genomics in salt tolerance between Arabidopsis and Arabidopsis‐related halophyte salt cress using Arabidopsis microarray. Plant Physiol 135: 1697–1709 15247402 10.1104/pp.104.039909 PMC519083

[ppl13166-bib-0093] Uozumi N , Kim EJ , Rubio F , Yamaguchi T , Muto S , Tsuboi A , Bakker EP , Nakamura T , Schroeder JI (2000) The Arabidopsis HKT1 gene homolog mediates inward Na(+) currents in *Xenopus laevis* oocytes and Na(+) uptake in *Saccharomyces cerevisiae* . Plant Physiol 122: 1249–1259 10759522 10.1104/pp.122.4.1249 PMC58961

[ppl13166-bib-0094] Vera‐Estrella R , Barkla BJ , Garcia‐Ramirez L , Pantoja O (2005) Salt stress in *Thellungiella halophila* activates Na^+^ transport mechanisms required for salinity tolerance. Plant Physiol 139: 1507–1517 16244148 10.1104/pp.105.067850 PMC1283785

[ppl13166-bib-0095] Véry A‐A , Nieves‐Cordones M , Daly M , Khan I , Fizames C , Sentenac H (2014) Molecular biology of K+ transport across the plant cell membrane: what do we learn from comparison between plant species? J Plant Physiol 171: 748–769 24666983 10.1016/j.jplph.2014.01.011

[ppl13166-bib-0096] Volkov V (2015) Salinity tolerance in plants. Quantitative approach to ion transport starting from halophytes and stepping to genetic and protein engineering for manipulating ion fluxes. Front Plant Sci 6: 873 26579140 10.3389/fpls.2015.00873 PMC4621421

[ppl13166-bib-0097] Volkov V , Wang B , Dominy PJ , Fricke W , Amtmann A (2004) Thellungiella halophila, a salt‐tolerant relative of *Arabidopsis thaliana*, possesses effective mechanisms to discriminate between potassium and sodium. Plant Cell Environ 27: 1–14

[ppl13166-bib-0098] Wang TT , Ren ZJ , Liu ZQ , Feng X , Guo RQ , Li BG , Li LG , Jing HC (2014) SbHKT1;4, a member of the high‐affinity potassium transporter gene family from *Sorghum bicolor*, functions to maintain optimal Na(+) /K(+) balance under Na(+) stress. J Integr Plant Biol 56: 315–332 24325391 10.1111/jipb.12144

[ppl13166-bib-0099] Wang WY , Liu YQ , Duan HR , Yin XX , Cui YN , Chai WW , Song X , Flowers TJ , Wang SM (2020) SsHKT1;1 is coordinated with SsSOS1 and SsNHX1 to regulate Na^+^ homeostasis in *Suaeda salsa* under saline conditions. Plant and Soil 449: 117–131

[ppl13166-bib-0100] Wong CE , Li Y , Labbe A , Guevara D , Nuin P , Whitty B , Diaz C , Golding GB , Gray GR , Weretilnyk EA , Griffith M , Moffatt BA (2006) Transcriptional profiling implicates novel interactions between abiotic stress and hormonal responses in *Thellungiella*, a close relative of *Arabidopsis* . Plant Physiol 140: 1437–1450 16500996 10.1104/pp.105.070508 PMC1435811

[ppl13166-bib-0101] Wu HJ , Zhang Z , Wang JY , Oh DH , Dassanayake M , Liu B , Huang Q , Sun HX , Xia R , Wu Y , Wang YN , Yang Z , Liu Y , Zhang W , Zhang H , Chu J , Yan C , Fang S , Zhang J , Wang Y , Zhang F , Wang G , Lee SY , Cheeseman JM , Yang B , Li B , Min J , Yang L , Wang J , Chu C , Chen SY , Bohnert HJ , Zhu JK , Wang XJ , Xie Q (2012) Insights into salt tolerance from the genome of *Thellungiella salsuginea* . Proc Natl Acad Sci U S A 109: 12219–12224 22778405 10.1073/pnas.1209954109 PMC3409768

[ppl13166-bib-0102] Yadav NS , Shukla PS , Jha A , Agarwal PK , Jha B (2012) The SbSOS1 gene from the extreme halophyte Salicornia brachiata enhances Na^+^ loading in xylem and confers salt tolerance in transgenic tobacco. BMC Plant Biol 12: 188 23057782 10.1186/1471-2229-12-188 PMC3548769

[ppl13166-bib-0103] Yang Y , Zhang C , Tang RJ , Xu HX , Lan WZ , Zhao F , Luan S (2019) Calcineurin B‐like proteins CBL4 and CBL10 mediate two independent salt tolerance pathways in Arabidopsis. Int J Mol Sci 20: 2421 10.3390/ijms20102421 PMC6566158 31100786

[ppl13166-bib-0104] Yang Z , Wang C , Xue Y , Liu X , Chen S , Song C , Yang Y , Guo Y (2019b) Calcium‐activated 14‐3‐3 proteins as a molecular switch in salt stress tolerance. Nat Commun 10: 1199 30867421 10.1038/s41467-019-09181-2 PMC6416337

[ppl13166-bib-0105] Yao X , Horie T , Xue S , Leung HY , Katsuhara M , Brodsky DE , Wu Y , Schroeder JI (2010) Differential sodium and potassium transport selectivities of the rice OsHKT2;1 and OsHKT2;2 transporters in plant cells. Plant Physiol 152: 341–355 19889878 10.1104/pp.109.145722 PMC2799368

[ppl13166-bib-0106] Yin X , Xia Y , Xie Q , Cao Y , Wang Z , Hao G , Song J , Zhou Y , Jiang X (2020) The protein kinase complex CBL10‐CIPK8‐SOS1 functions in Arabidopsis to regulate salt tolerance. J Exp Bot 71: 1801–1814 31858132 10.1093/jxb/erz549 PMC7242078

[ppl13166-bib-0107] Yu L , Nie J , Cao C , Jin Y , Yan M , Wang F , Liu J , Xiao Y , Liang Y , Zhang W (2010) Phosphatidic acid mediates salt stress response by regulation of MPK6 in *Arabidopsis thaliana* . New Phytol 188: 762–773 20796215 10.1111/j.1469-8137.2010.03422.x

[ppl13166-bib-0108] Zhang M , Cao Y , Wang Z , Wang ZQ , Shi J , Liang X , Song W , Chen Q , Lai J , Jiang C (2018) A retrotransposon in an HKT1 family sodium transporter causes variation of leaf Na(+) exclusion and salt tolerance in maize. New Phytol 217: 1161–1117 29139111 10.1111/nph.14882

[ppl13166-bib-0109] Zhou H , Lin H , Chen S , Becker K , Yang Y , Zhao J , Kudla J , Schumaker KS , Guo Y (2014) Inhibition of the Arabidopsis salt overly sensitive pathway by 14‐3‐3 proteins. Plant Cell 26: 1166–1182 24659330 10.1105/tpc.113.117069 PMC4001376

[ppl13166-bib-0110] Zhu JK (2016) Abiotic stress signaling and responses in plants. Cell 167: 313–324 27716505 10.1016/j.cell.2016.08.029 PMC5104190

[ppl13166-bib-0111] Zorb C , Geilfus CM , Dietz KJ (2019) Salinity and crop yield. Plant Biol (Stuttg) 21(Suppl 1): 31–38 30059606 10.1111/plb.12884

